# Baseline demographics and disease characteristics of patients with episodic or chronic cluster headache: data from two phase 3 randomized clinical trials in Europe and North America

**DOI:** 10.3389/fneur.2023.1293163

**Published:** 2023-12-15

**Authors:** Rigmor Hoejland Jensen, Cristina Tassorelli, Tina M. Myers Oakes, Jennifer N. Bardos, Chunmei Zhou, Yan Dong, Sheena K. Aurora, James M. Martinez

**Affiliations:** ^1^Danish Headache Center, Department of Neurology, Rigshospitalet Glostrup, University of Copenhagen, Copenhagen, Denmark; ^2^Department of Brain and Behavioural Sciences, University of Pavia, Pavia, Italy; ^3^Headache Science and Neurorehabilitation Centre, IRCCS Mondino Foundation, Pavia, Italy; ^4^Eli Lilly and Company, Lilly Corporate Center, Indianapolis, IN, United States

**Keywords:** clinical trial, cluster headache, demographic analyses, disease characteristics, galcanezumab, prevention

## Abstract

**Objective:**

Two phase 3 galcanezumab trials were conducted in Europe and North America to analyze the reduction of weekly cluster headache (CH) attack frequency in populations with episodic and chronic CH. The current study aims to illustrate prospectively recorded baseline clinical data from these trials and to identify possible predictors of response.

**Methods:**

Patients (aged 18–65 years) met The International Classification of Headache Disorders 3rd edition-beta criteria for CH. Attacks were evaluated using an electronic headache diary for 7-day (episodic) or 14-day (chronic) eligibility assessments before patients were randomized 1:1 to monthly subcutaneous galcanezumab 300 mg or placebo.

**Results:**

Data were collected from 106 patients with episodic and 237 with chronic CH. Overall, the mean age [standard deviation] was 45.4 [11.0] years; patients were predominantly White (84.5%), male (75.8%), and European (77.6%). Patients with episodic CH reported 17.5 [10.0] attacks/week; patients with chronic CH reported 18.8 [10.2] attacks/week. The average pain severity score (range 0–4) was 2.5 [0.7] for episodic CH and 2.7 [0.7] for chronic CH. Higher attack frequency was a possible predictor of response to galcanezumab; potential negative predictors of response were greater attack severity and duration.

**Conclusion:**

This large dataset of patients with CH provides reliable systematically and prospectively collected information on disease characteristics. The analysis in episodic CH underscores potential predictors of response worth considering for future CH trial design.

**Clinical Trial Registration:**

ClinicalTrials.gov, identifiers: NCT02397473 and NCT02438826.

## 1 Introduction

Cluster headache (CH) can affect adults of all ages, with onset occurring at any time but usually at or before 30 years of age (1). The disorder is more prevalent in men than in women; approximately 70% of respondents to recent surveys of individuals with CH were male, and the male-to-female ratio in a recent study conducted at a specialized clinic was 2:1 (1–3).

Limited studies are available that include prospective recording of baseline CH features from randomized controlled trials (RCTs) with larger CH populations. When the studies herein initiated, two RCTs with approximately ≥100 patients had been published evaluating sumatriptan (*N* = 168) and sodium valproate (*N* = 96) in the prevention of CH (4, 5). The limited number of prospective studies on CH renders the design of clinical trials for this disease problematic, especially when inclusion and exclusion criteria must be clearly defined. This may be one reason for the persistently limited availability of effective drugs to treat this extremely severe and debilitating disease (6).

Until recently, only cross-sectional surveys were available in the literature for large CH populations. For example, in the United States Cluster Headache Survey, 80% of surveyed patients with CH had headaches daily, and the average number of attacks/day experienced by individuals with CH varied widely; however, the survey did not stratify these values as episodic cluster headache (ECH) or chronic cluster headache (CCH) types (1). The majority of survey respondents reported one to four attacks/day, and approximately 20% of respondents reported five to eight attacks/day (1). In comparison, respondents in the Danish Cluster Headache Study and the Cluster Headache Questionnaire international survey (two additional cross-sectional studies) reported mean averages of 3.6 attacks/day and 3.9 attacks/day, respectively (2, 7). More recently, the prospective, observational Korean Cluster Headache Registry study reported a median (interquartile range) daily attack frequency of 1.1 (1.0–3.0) among study participants with CH (8).

Galcanezumab is a humanized monoclonal antibody that binds to and inhibits calcitonin gene-related peptide (CGRP) activity (9–11), with demonstrated efficacy in migraine (12, 13). Two phase 3, prospective, double-blind, randomized, placebo-controlled studies (two of the largest CH trials completed) conducted in Europe and North America assessed the safety and efficacy of galcanezumab vs. placebo in reducing weekly attack frequency in patients with ECH (NCT02397473) and CCH (NCT02438826) (14, 15). The primary outcome measure and key secondary measure (weekly frequency of CH attacks and 50% response, respectively) were reached in the ECH study, while the CCH was a negative study. Together, the two trials enrolled a large patient population with a definite diagnosis of ECH or CCH and used electronic diaries to collect prospective data on CH attacks for 7 or 14 days, respectively, before patients were randomized to interventional treatment (14, 15).

This report has two objectives: to elucidate prospectively recorded disease characteristics from the largest select population of patients with ECH or CCH to date and to perform a *post hoc* analysis of the ECH trial in an attempt to identify potential predictors of response to galcanezumab.

## 2 Methods and materials

### 2.1 Study design and patients

**Figure 1:**
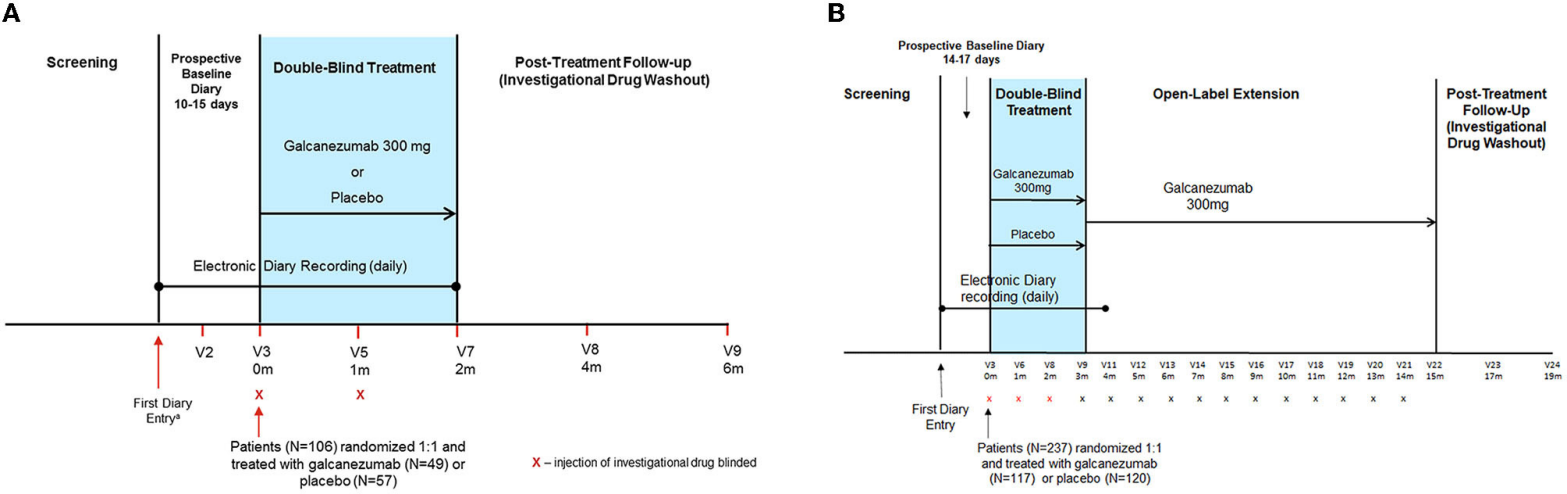
Study designs for the **(A)** episodic CH and **(B)** chronic CH studies. The ECH study comprised four phases: screening/washout phase; a prospective baseline phase; an 8-week double-blind treatment phase; and a 16-week post-treatment follow-up (washout) phase. The CCH study comprised five phases: screening/washout phase; a prospective baseline phase; a 12-week, double-blind treatment phase; a 1-year, open-label, extension phase; and a 16-week, post-treatment follow-up (washout) phase. For each patient, the prospective baseline phase began on the day the patient first recorded a CH attack in their electronic diary. m, month; V, visit number.

Details of the study designs, objectives, and endpoints of the two trials in populations with episodic and chronic CH have been published previously (14, 15). The ECH study was a phase 3, randomized (1:1), double-blind, placebo-controlled trial of subcutaneous (SC) galcanezumab 300 mg administered once monthly in patients with ECH (14). It comprised four study phases (Figure 1A): screening; prospective baseline (10 to 15 days, of which 7 consecutive days were used to assess eligibility and capture disease characteristics); double-blind, placebo-controlled treatment (8 weeks); and post-treatment follow-up (16 weeks) (14).

The CCH study was a phase 3, multicenter, randomized (1:1), double-blind, placebo-controlled trial of SC galcanezumab 300 mg administered once monthly in patients with CCH (15). It comprised five study phases (Figure 1B): screening, prospective baseline (14 to 17 days, of which 14 consecutive days were used to assess eligibility and capture disease characteristics), double-blind treatment (12 weeks), open-label treatment (optional 1-year), and post-treatment follow-up (investigational drug washout; 16 weeks) (15).

These two studies enrolled patients in Europe and North America who were aged 18 to 65 years and who met The International Classification of Headache Disorders 3rd edition-beta (16) diagnostic criteria for either ECH or CCH, respectively (14, 15). Key exclusion criteria were the same for the two studies and included current enrollment in another clinical trial; current or previous use of CGRP or nerve growth factor antibodies; suspected presence of another distinct trigeminal autonomic cephalalgia; and lifetime history of migraine variants that could implicate or be confused with ischemia. Patients with serious or unstable medical conditions that would preclude study participation (including but not limited to pregnancy, significant risk of suicide, history of substance abuse, or dependence within the past year, history of stroke, intracranial or carotid aneurysm, recent history of cardiovascular events, or risk for serious cardiovascular events including abnormal electrocardiogram findings) were also excluded. Study patients were allowed concomitant medications for acute or abortive treatment of CH, including high-flow oxygen, oral triptans, sumatriptan SC injections, sumatriptan nasal spray, zolmitriptan nasal spray, acetaminophen, and non-steroidal anti-inflammatory drugs (NSAIDs).

Concomitant preventive medications were not permitted in the ECH study. In contrast, up to six concomitant preventive medications were allowed in the CCH study (verapamil ≤ 480 mg/day, lithium, melatonin, valproate, gabapentin, and topiramate) if the patient was on a stable dose for 2 months prior to the prospective baseline and remained on a stable dose during the double-blind period.

These studies were conducted in accordance with the International Conference on Harmonization Guidelines for Good Clinical Practice and the Declaration of Helsinki and approved by each institution's ethical review board. Patients provided written informed consent before enrollment. Each study was registered with ClinicalTrials.gov; identifiers NCT02397473 and NCT02438826, respectively.

### 2.2 Data collection

In both the ECH and CCH studies, patients recorded daily CH attack information and acute medication use in an electronic patient-reported outcome diary during the prospective baseline (14, 15). The following patient demographics and characteristics were recorded at screening for all intent-to-treat patients: age, sex, race, ethnicity, country, region, body mass index, baseline alcohol, tobacco, caffeine and nicotine use and consumption, medical history and pre-existing conditions, lifetime suicidal ideation and behavior, and use of preventive and/or concomitant medications. Data collected during the prospective baseline phase were used to establish the baseline frequency of CH attacks, attack duration, attack severity, and acute/abortive medication use. In the CCH study, data from the electronic case report form were used to evaluate the use of preventive medications (when allowed).

Patients recorded the number of CH attacks, attack severity/duration, and acute/abortive medications in their electronic patient-reported outcome diary during the prospective baseline and double-blind treatment phases (and through the 1^st^ month of the open-label treatment phase for chronic CH). This report focuses on data from the electronic patient-reported outcome diaries from the prospective baseline phase. Information regarding abortive medication use, average CH attack duration, and average CH attack pain severity was also recorded. Regardless of acute medication use, average pain severity during the previous 24-h period was rated using a 5-point pain scale, where 0 = no pain, 1 = mild pain, 2 = moderate pain, 3 = severe pain, and 4 = very severe pain (17). To determine attack duration, patients answered the following query each day in their electronic patient-reported outcome diary: on average, what was the duration of your CH attack(s) during this 24-h period? Patients were instructed to round up if their average duration was between two of the following choices: 15 min, 30 min, 1 h, 2 h, 3 h, and >3 h. If the >3-h choice was selected, 4 h was imputed for that 24-h period. To calculate the total daily (24-h) duration, the number of attacks was multiplied by the average duration for that day. If the calculated total daily duration was >24 h, it was set to 24 h.

### 2.3 Statistical analyses

All analyses for baseline demographic and disease state characteristics were conducted on intent-to-treat patients who were randomly assigned to a treatment group and received at least one dose of study drug. Medical history and pre-existing conditions were summarized by preferred term. Categorical variables were summarized using frequency and percent while continuous variables were summarized using mean and standard deviation (SD). All statistical analyses except responder analyses were descriptive in nature, and no formal statistical inferences were conducted. A *post hoc* multiple logistic regression model was conducted to evaluate the association between the 50% response (yes or no) at Week 3 and selected demographic (age ≥40 years, European residence, and use of oxygen or SC sumatriptan) and prospective baseline disease characteristics (weekly attack frequency, attack severity, attack duration, and categorical attack frequency ≤ 4) in the ECH study. The impact of each covariate on the odds ratio (OR; 95% confidence interval) of achieving response vs. non-response was reported for the galcanezumab-treatment group (35 responders and 11 non-responders). Because the CCH study was a negative study, a 50% responder analysis was not performed. All analyses were performed using SAS^®^ software, version 9.4.

## 3 Results

### 3.1 Baseline demographics

In total, 343 patients were randomized and treated: 106 with ECH (57 placebo- and 49 galcanezumab-treated) and 237 with CCH (120 placebo- and 117 galcanezumab-treated). Overall, the mean age (SD) was 45.4 (11.0) years; patients in both studies were predominantly White (*n* = 290 [84.6%]), from Europe (*n* = 266 [77.6%]), and male (*n* = 260 [75.8%]) (Supplementary Table 1). The mean (SD) age of patients with ECH was 46.4 (11.1) years (69.8% were ≥40 years of age), and 18/106 (17.0%) were female. Regarding racial classification, 6/106 (5.7%) patients with ECH were Black or African American and 10/106 (9.4%) were classified as “other.” Patients with CCH had a mean age of 45.0 (10.9) years (70.9% were ≥40 years of age) and 65/237 (27.4%) were female; 2/237 (0.8%) were Black or African American and 35/237 (14.8%) were classified as “other.”

Thirty-six of 106 patients with ECH (32.1%) were from the United States and 70/106 (67.9%) were from Europe. Thirty-three of 237 patients with CCH (13.9%) were from the United States and 204/237 (86.1%) were from Europe.

### 3.2 Baseline disease state characteristics collected at Visit 1

Patients with ECH reported a history of CH illness for an average of 16.8 years, more than double the average reported history for patients with CCH (8.0 years). Despite having a shorter disease history, patients with CCH exhibited suicidal ideation/behavior more frequently (Supplementary Table 1). Concomitant migraine preventive treatments were not allowed in the ECH study, whereas in the CCH study up to six preventive treatments were allowed and 150/237 patients (63.3%) were taking ≥1 preventive treatment; of these, 108/150 (72.0%) used one preventive treatment and 36/150 (24.0%) used two (15). Verapamil was the most common preventive drug (49.8%), followed by lithium (13.1%); all other preventives were each used by <10% of patients (15, 18).

### 3.3 Disease state characteristics collected at prospective baseline

Disease state characteristics collected during the prospective baseline phase are shown in Table 1. Patients in both studies reported a similar mean number of weekly CH attacks (ECH: 17.5 ± 10.0; CCH: 18.8 ± 10.2). The mean duration of weekly total attacks was 15.5 h (min, max: 2.5, 88.0) for patients with ECH and 18.3 h (min, max: 1.5, 139.5) for patients with CCH. The most common reported daily attack frequency was ≤ 2/day in both studies (ECH: 49.1%; CCH: 42.6%), followed by >2–4 attacks/day (ECH: 36.8%; CCH: 40.5%). Less than 20% of patients reported >4 attacks/day (ECH: 14.2%; CCH: 16.9%) (Table 1). The average pain severity of CH attacks was moderate to severe (ECH: 2.5; CCH: 2.7).

**Table 1:** Prospective baseline disease characteristics collected during the prospective baseline phase.

	**ECH total (14) *N =* 106**	**CCH total (15) *N =* 237**
Number of weekly CH attacks		
Mean (SD)	17.5 (10.0)	18.8 (10.2)
Median	15.0	16.2
Min, Max	4.0, 51.0	5.0, 47.5
Average severity of CH pain^a^ for CH attack, days	
Mean (SD)	2.5 (0.7)	2.7 (0.7)
Min, Max	1.0, 4.0	1.1, 4.0
Average daily CH attack frequency, *n* (%)		
>4 attacks per day	15 (14.2)	40 (16.9)
>2 to 4 attacks per day	39 (36.8)	96 (40.5)
≤ 2 attacks per day	52 (49.1)	101 (42.6)
Weekly total CH attack duration, hours		
Mean (SD)	15.5 (14.5)	18.3 (18.7)
Min, Max	2.5, 88.0	1.5, 139.5

CCH, chronic cluster headache; CH, cluster headache; ECH, episodic cluster headache; *N*, number of intent-to-treat patients with non-missing demographic measures; n, number of patients within each specific category; SD, standard deviation. ^a^Pain severity rated using a 5-point pain scale: 0 = no pain, 1 = mild pain, 2 = moderate pain, 3 = severe pain, and 4 = very severe pain (17). There were no significant differences in disease characteristics between placebo- and galcanezumab-treatment groups within either study.

During the prospective baseline, the acute treatments used by the greatest percentages of both patients with ECH and those with CCH to treat their CH attacks were SC sumatriptan (ECH: 52.8%; CCH: 62.9%) and oxygen (ECH: 50.9%; CCH: 59.1%) (Supplementary Table 2). The mean weekly number of times using SC sumatriptan (ECH: 9.1; CCH: 9.3) and oxygen (ECH: 15.2; CCH: 16.4) was similar in the two studies and the mean total weekly dose for SC sumatriptan was 49.5 mg for ECH and 55.8 mg for CCH (data not shown).

### 3.4 Alcohol, caffeine, nicotine, and tobacco use and consumption

Data were collected at Visit 1 regarding patients' reported alcohol, caffeine, nicotine, and tobacco use (Supplementary Table 3). Current caffeine use was similarly high in the two studies and more than half of patients with ECH reported current alcohol use. Nicotine use was similarly low in the two studies while more than half of patients with CCH reported tobacco use. Among current tobacco users in the two studies, the mean daily consumption of different tobacco types suggests that smoking (rather than the use of smokeless tobacco, also known as chewing tobacco) constituted the vast majority of tobacco consumption regardless of CH type (ECH: 100% and CCH: 99.4% of mean daily current tobacco consumption, respectively). Fewer than 5% of patients in either study reported current nicotine use, defined as e-cigarettes, transdermal nicotine patches, or gum.

### 3.5 Pre-existing conditions

All patients in both studies reported ≥1 pre-existing condition. Pre-existing conditions reported by ≥5% of patients in either study are reported in Supplementary Table 4. The most frequently reported conditions (>10%) were insomnia in both the ECH and CCH studies, and gastroesophageal reflux disease in the ECH study and hypertension in the CCH study. Hypertension was also present in the ECH study (6.6%). In addition to the hypertension and hypercholesterolemia reported in both studies, the prevalence of other pre-existing conditions related to cardiovascular risk included type 2 diabetes mellitus (ECH: 0.9%; CCH: 0.4%) and dyslipidemia (ECH: 3.8%; CCH: 1.7%) (data not shown).

### 3.6 ECH responders vs. non-responders

The results of the responder analysis for selected demographic and CH disease characteristics collected prospectively are shown in Table 2. The likelihood of being a responder decreased in patients who were 40 years or older (OR = 0.511), of European residence (OR = 0.664), using oxygen or SC sumatriptan (OR = 0.3), and reporting ≤ 4 attacks/day (OR = 0.045). However, the likelihood of being a responder increased as weekly attack frequency increased (OR = 1.082), consistent with the reduced likelihood of being a responder in patients with ≤ 4 CH attacks/day. While the ORs suggested trends toward being a responder or non-responder, none of these results were statistically significant.

**Table 2:** ECH responders vs non-responders to galcanezumab: prospective baseline disease characteristics.

**Characteristics**	**Responders *N =* 35**	**Non-responders *N =* 11**	**Odds ratio (OR) responder vs. non-responder (95% CI)**
Age ≥40 years, *n* (%)	24 (68.6)	9 (81.8)	0.511 (0.076, 3.458)
European region, *n* (%)	23 (65.7)	9 (81.8)	0.664 (0.075, 5.887)
Use of oxygen or SC sumatriptan, *n* (%)	28 (80.0)	10 (90.9)	0.3 (0.021, 4.368)
Weekly CH attacks, mean (SD)	17.7 (9.6)	16.3 (8.4)	1.082 (0.937, 1.251)
Average severity of CH pain for CH attack days, mean (SD)	2.4 (0.7)	2.6 (0.7)	0.617 (0.182, 2.093)
Weekly total CH attack duration, mean (SD), hours	13.4 (11.2)	19.2 (23.6)	0.918 (0.821, 1.027)
Average daily CH attack frequency, ≤ 4 attacks/day, n (%)	29 (82.9)	10 (90.9)	0.045 (< 0.001, 112.672)

CH, cluster headache; CI, confidence interval; ECH, episodic cluster headache; N, number of intent-to-treat patients with non-missing demographic measures; n, number of patients within each specific category or number of intent-to-treat patients; n, number of patients with non-missing demographic measures; SC, subcutaneous; SD, standard deviation. For continuous variables, if OR >1, the likelihood of being a responder (vs. non-responder) increases as the continuous parameter increases. Otherwise, if OR <1, the likelihood of being a responder decreases as the parameter increases. For the categorical variables, if OR <1, the likelihood of being a responder decreases in the reported category for the specific parameter. Otherwise, if OR >1, the likelihood of being a responder increases in the reported category. For the calculation of average pain severity of CH attack, pain severity is categorized on a 5-point scale: 0 = no pain, 1 = mild pain, 2 = moderate pain, 3 = severe pain, and 4 = very severe pain.

Demographics and baseline characteristics collected at Visit 1 were generally similar between responders and non-responders in galcanezumab-treated patients, with most patients in both groups being White males aged 40 and older and from Europe. However, all patients reporting a history of suicidality were in the responder group (Table 3). During the prospective baseline, the CH disease characteristics suggesting a difference, on average, between ECH responders, and non-responders among galcanezumab-treated patients were attack frequency, attack duration, and attack severity (Table 2).

**Table 3:** ECH responders vs. non-responders to galcanezumab: baseline demographics and disease characteristics collected at Visit 1.

**Demographic variable**	**Responders**	**Non-responders**
	***N** =* **35**	***N** =* **11**
Mean (SD) age, years	47.5 (11.3)	45.1 (9.1)
Age group, *n* (%)		
< 40	11 (31.4)	2 (18.2)
≥40	24 (68.6)	9 (81.8)
Males, *n* (%)	30 (85.7)	9 (81.8)
Race, *n* (%)		
Black or African American	1 (2.9)	0 (0.0)
White	30 (85.7)	11 (100.0)
Other	4 (11.4)	0 (0.0)
Mean (SD) BMI, kg/m^2^	25.8 (4.4)	27.5 (3.6)
Region, *n* (%)		
Europe	23 (65.7)	9 (81.8)
North America	12 (34.3)	2 (18.2)
Mean (SD) duration of cluster headache illness, years	15.8 (10.0)^a, b^	14.2 (11.4)
Lifetime suicidal ideation prior to screening,^c^ *n* (%)	9 (25.7)	0 (0.0)
Lifetime suicidal behavior prior to screening,^d^ *n* (%)	1 (2.9)	0 (0.0)

BMI, body mass index; C-SSRS, Columbia-Suicide Severity Rating Scale; ECH, episodic cluster headache; SD, standard deviation. ^a^*N* = 33. ^b^Duration of cluster headache illness (years) is defined as (informed consent date – first cluster headache medical history start date + 1)/365.25. ^c^Suicidal ideation includes a “yes” answer to any of the five suicidal ideation questions (categories 1–5) on the C-SSRS. ^d^Suicidal behavior includes a “yes” answer to any of the five suicidal behavior questions (categories 6–10) on the C-SSRS.

## 4 Discussion

### 4.1 Significance of present data in the context of other clinical trial populations

These two studies in CCH and ECH were conducted in Europe and North America. Patients in the CCH study reported a shorter history of illness than those in the ECH study and the CCH population contained more women than the ECH population. Baseline demographic characteristics in these two studies were similar to those described by more recent controlled studies (ACT-1 and ACT-2) in the acute treatment of both ECH and CCH (Table 4) (19, 20). It is also informative to compare these galcanezumab CH studies with older, controlled CH prevention studies (Table 4). In comparison with a randomized, double-blind study evaluating the ability of verapamil vs. placebo to prevent attacks in people with ECH (21), the present ECH study population had a numerically smaller percentage of male patients (83 vs. 90%) and similarities are noted in patients' mean ages (46.4 years vs. 43–44 years) and mean durations of CH illness (16.8 years vs. 15–16 years). Compared with a double-blind, crossover comparison study assessing the ability of verapamil vs. lithium to prevent attacks in patients with CCH (22), the CCH population in the present study also had a smaller percentage of male patients (73 vs. 90%) and patients were similar in age (45 years vs. 43 years), with comparable mean durations of CH illness (8 years vs. 9 years). The smaller percentage of male patients in the more recent CCH study is consistent with reports of a trend toward increased awareness of the occurrence of CH in women and/or decreasing male predominance among patients with CH (3, 23).

**Table 4:** Baseline characteristics of patients with CH in selected clinical trials.

**Parameter**	**Goadsby et al. [ACT-2] (19)**	**Silberstein et al. [ACT-1] (20)**	**El Amrani et al. (5)^a^**	**Leone et al. (21)^b^**	**Monstad et al. (4)^a^**	**Bussone et al. (22)^c^**
Study type	Randomized, double-blind, sham-controlled (non-invasive vagus nerve stimulation vs. sham) *N =* 102	Randomized, double-blind, sham-controlled (non-invasive vagus nerve stimulation vs. sham) *N =* 150	Randomized, double-blind, placebo-controlled study (sodium valproate vs. placebo) *N =* 96	Randomized, double-blind, double-dummy, parallel-group (verapamil vs. placebo) *N =* 30	Randomized, double-blind, placebo-controlled study (sumatriptan vs. placebo) *N =* 168	Randomized, double-blind, double-dummy, crossover comparison study (verapamil vs. lithium) *N =* 30
Mean (SD) age for ECH, y	42.9 (12.7)	48.4 (12.5)	Placebo: 43.6 (11.5) Valproate 47.0 (11.3)	Placebo: 43 (10) Verapamil: 44 (8)	40 (10)	-
Mean (SD) age for CCH, y	46.5 (9.6)	46.8 (13.0)		-		43 (11)
% Male ECH	73%	83%	NR	Placebo: 93% Verapamil: 87%	NR	-
% Male CCH	71%	86%	NR	-	NR	90%
Mean (SD) duration of CH illness, y	NR	NR	NR	Placebo: 15 (10) Verapamil: 16 (11)	NR	9 (5)
ECH attack frequency at baseline	10 (1, 53)^d^	NR	Placebo: 12.0 (6.4)^e^ Valproate: 12.1 (6.3)^e^	Placebo: 1.4 (0.8)^f^ Verapamil: 1.9 (0.9)^f^	2^g^	-
CCH attack frequency at baseline	11 (2, 39)^d^	NR		-		NR

^a^Study did not present data for patients with ECH and CCH separately. ^b^Study included patients with ECH only. ^c^Study included patients with CCH only. ^d^Median (min, max) weekly attack frequency recorded prospectively. ^e^Mean (SD) weekly attack frequency recorded prospectively. ^f^Mean (SD) daily attack frequency recorded prospectively. ^g^Median daily attack frequency for both treatment groups and overall. Min and max were not provided. CCH, chronic cluster headache; CH, cluster headache; ECH, episodic cluster headache; NR, parameter not reported.

### 4.2 Significance of present data in the context of previous data from surveys and population-based studies

Surveys and population-based CH studies can vary widely in reporting demographic and disease characteristics. For example, more recent Western studies report mean ages and male-to-female ratios similar to the two studies reported here (2, 7, 24, 25); in contrast, recently published data from the Korean Cluster Headache Registry study describe a Korean patient population that is comparatively younger with a larger male-to-female ratio (8). All of the studies presented for comparison tended not to report the distribution of ECH and CCH subtypes or separate demographics for subtypes (Table 5).

**Table 5:** Baseline characteristics of patients with CH in selected surveys and population-based studies.

**Parameter**	**Lee et al. (8)**	**Lund et al. (7)**	**Joshi et al. (26)**	**Rozen et al. (1)**	**Schurks and Diener (27)**
Study type	Prospective, observational study	Observational, case-controlled, questionnaire-based survey	Observational, case-controlled, retrospective, population-based database study	Observational survey	Review of selected literature
Mean (SD) age, y	37.8 (10.7)	46.2 (11.5)	43.4 (NR)	Age distribution: < 20 years: 0.6% 21–30 years: 12% 31–40 years: 27% 41–50 years: 34% 51–60 years: 21% 61+ years: 5%	NR
% Male	84%	67%	80%	72%	Varied among reviewed studies from 0 to 100%
Current tobacco use	NR	Smoking: 48%	Smoking: 68%	Current or prior tobacco smoking or chewing: 73%	Smoking: 21–94%
Alcohol use	NR	Intake yes: 61% Unhealthy intake: 11% Damaging intake: 5%	Moderate: 1% Abuse: 17%	Intake yes: ~65% Diagnosed alcoholic: 3%	Regular consumers: 43–91%
Lifetime suicidal ideation at baseline	NR	NR	NR	55%	N/App
Lifetime suicidal behavior at baseline	NR	NR	0%	2%	N/App
Daily CH attack frequency at baseline	Median (IQR): 1.1 (1.0–3.0)	Mean (SD): 3.6 (2.3)	NR	Attacks per day: 1 attack: 22% 2 attacks: 24% 3 attacks: 18% 4 attacks: 12% 5–8 attacks: 20%	NR

CH, cluster headache; IQR, interquartile range; N/App, not applicable; NR, parameter not reported. The studies shown included both patients with chronic and episodic CCH and did not present parameters according to CH type.

The percentages of patients reporting current tobacco smoking in these ECH and CCH studies (52 and 63%, respectively) were consistent with those reported in other studies (1, 7, 14, 15) but much higher than the 2016 global average reported by the World Health Organization (22%) (28). Rates of current alcohol use in these studies were 61% among patients with ECH and 47% among those with CCH. In comparison, the U.S. Cluster Headache Survey study of individuals with CH reported an alcohol intake rate of ~65% (1), while 61% (71% in ECH and 43% in CCH) of respondents in the Danish Cluster Headache Study consumed alcohol (7) (Table 5).

Reporting of suicidal ideation or behavior is inconsistent across CH publications. In one survey-based study, 55% of respondents reported lifetime suicidal ideation (1), which far exceeds the frequencies reported in these ECH (13%) and CCH (23%) studies (14, 15), although the frequency of suicidal behavior was similar. This difference in the frequency of suicidal ideation could be due to differences in how data were collected. Compared to a self-reported survey, the present studies used the rater-based Columbia-Suicide Severity Rating Scale and patients may have felt more comfortable reporting ideation via a survey.

The most common comorbidities observed at baseline in the present studies are generally consistent with data reported previously (1, 25, 26). Depression and anxiety were reported by patients in both ECH and CCH studies, albeit somewhat more frequently in patients with CCH; insomnia was common in both studies. These psychiatric comorbidities are also frequently reported in other studies, but without differentiation between CH subtypes (25, 26, 29). Although generally consistent, several conditions related to cardiovascular risk were reported at baseline in the current trials at slightly lower rates than in surveys or population-based studies (1, 7, 26). The variation in these comorbidities compared with previous literature may be due to differences in the methods and standards used to identify comorbid conditions (Medical Dictionary for Regulatory Activities preferred term vs. International Classification of Diseases, Ninth Revision criteria) (26), or in collection methods (insurance claims database vs. medical records or survey questionnaires) (1, 7, 26).

### 4.3 Significance of disease characteristics collected prospectively

The prospective baseline collection of patient-reported CH attack variables and use of acute medications from these ECH and CCH studies provides important longitudinal data for future clinical trial planning in CH. Prospective reporting of CH disease characteristics in RCTs is not universally adopted. A recent review of peer-reviewed publications of preventive RCTs identified nine trials that reported prospective baseline data and seven of these enrolled ≤ 34 patients (6). In the current ECH and CCH studies, the frequency of weekly CH attacks and average daily attack frequency were similar. In contrast, average attack duration and average attack pain severity were numerically higher in the CCH study (Table 1). Prospective data reported in studies vary in the type and amount included, as shown in Table 4, limiting comparisons across all the variables reported prospectively in the current studies. In general, the number of daily attacks when reported prospectively ranged from one to three, consistent with current studies (4, 8, 21, 30, 31). Interestingly, a lower prospective weekly attack frequency was reported in two prevention studies that failed to meet their primary endpoints: the sodium valproate study by El Amrani et al. (5) (Table 4) and a more recent ECH prevention study of fremanezumab that reported a weekly average of 12.7 to 13.3 CH attacks across treatment groups (32). Similar prospective mean weekly attack frequencies of approximately 15 and 18 were reported in two small studies of 11 patients each with ECH and refractory CCH, respectively (33, 34).

During the prospective baseline, the majority of patients with ECH and CCH reported using SC sumatriptan and oxygen, which was consistent with the higher weekly number of times each of these medications were used compared to other acute treatments (Supplementary Table 2). Patients in the ECH study reported greater use of analgesics such as acetaminophen/paracetamol and NSAIDs compared to CCH and use of oral and intranasal triptans was reported less frequently in both studies (14, 15). However, it is noted that oral triptans were not allowed in the original protocol, but were added in a later amendment, limiting the interpretation of their actual use. Oxygen use in our ECH study was less common than in the cohort of patients with ECH in ACT-2, but more common in our CCH study than among patients with CCH in ACT-2 (14, 15, 19).

### 4.4 Potential predictors of response

Potential predictors of response to galcanezumab were evaluated in the current ECH study, given the study met its primary endpoint by demonstrating a significant mean weekly attack reduction of 3.5 over placebo (*p* = 0.04) and a significantly greater percentage of patients with a 50% or greater reduction in attack frequency (galcanezumab: 71%, placebo: 53%; *p* = 0.046). A *post hoc* analysis of selected demographic and disease state characteristics reported during the prospective baseline period suggested that age ≥40 years, European residence, oxygen or SC sumatriptan use, ≤ 4 CH attacks/day, higher average CH pain severity, and longer average CH attack duration were negative predictors of response, with a stronger association for oxygen or SC sumatriptan use, age ≥40 years, and ≤ 4 attacks/day. The analysis also suggests a higher weekly CH attack frequency is associated with response (Table 2). While these data must be interpreted with caution due to their *post hoc* nature and small sample sizes, it is worth noting a higher pain intensity has been reported previously as a predictor of non-response to treatment in patients with medication overuse headache (35, 36). Thus, our data need to be confirmed in larger studies of representative and homogenous patient populations where the diurnal pattern is also included, given that nocturnal attacks are reported to be clinically more severe and longer lasting.

### 4.5 Limitations and strengths

The main limitation of these studies is the rigorous inclusion/exclusion criteria, such as exclusion of patients with serious or unstable medical conditions that would interfere with study participation, including recent acute cardiovascular events; however, patients with other comorbid diseases were included. Another limitation is that data were not collected regarding whether patients with CCH were newly diagnosed with CCH or transformed from a previous ECH diagnosis; such information might shed additional light on the increased rate of suicidality in patients with CCH. Data on prior bout duration were also not collected, although in the ECH study the duration of prior bouts was required to be ≥6 weeks, limiting the analytic comparisons that may have been possible. While consistent with the guidelines for controlled trials in CH, the current ECH study did not allow any preventive medications (37); however, this prohibition may have limited the voluntary participation of patients who had a positive treatment response from available preventive medications. That the two studies were conducted solely in Europe and North America limits generalizability of the findings because several CH characteristics differ between Asian and European/North American patients (38).

Finally, the findings from the responder analysis should be interpreted with caution due to the small sample size of the ECH study (*n* = 46 patients: 35 responders and 11 non-responders). Because the CCH study was a negative study, a responder analysis was not conducted to evaluate the association between response status and baseline disease characteristics.

The main strength of the data presented here is represented by the prospective evaluation of headache characteristics collected using a timestamp electronic diary from a large population spanning multiple countries. Furthermore, the clinical expertise of the enrolling centers strengthens confidence in the correctness of diagnoses compared to survey-based studies. The fact that the demographic data and disease characteristics of this population at baseline were similar to those in prior published studies using various sampling methods suggests that, although highly selected, our study population may be considered representative of the clinical population with CH in Europe and North America.

## 5 Conclusion

The two phase 3, placebo-controlled studies evaluating galcanezumab for CH detailed herein provided the ability to record baseline demographics and clinical characteristics precisely, together with the prospective daily collection of CH attack characteristics and acute medication use in a relatively large population with ECH and CCH. The analysis in patients with ECH underscores potential predictors of response worth considering in the design of future CH trials.

## Data availability statement

Eli Lilly and Company provides access to all individual participant data collected during the trial, after anonymization, with the exception of pharmacokinetic or genetic data. Data are available to request 6 months after the indication studied has been approved in the US and EU and after primary publication acceptance, whichever is later. No expiration date of data requests is currently set once data are made available. Access is provided after a proposal has been approved by an independent review committee identified for this purpose and after receipt of a signed data sharing agreement. Data and documents, including the study protocol, statistical analysis plan, clinical study report, and blank or annotated case report forms, will be provided in a secure data sharing environment. For details on submitting a request, see the instructions provided at www.vivli.org.

## Ethics statement

The studies involving humans were approved by the appropriate institutional or ethical review board for each site, as follows: United States: Quorum Review, Inc., Seattle, WA; Stanford University Hospital, Palo Alto, CA; Thomas Jefferson University, Office of Human Research, Philadelphia, PA; Mayo Clinic of Scottsdale, Rochester, MN; University of Texas Southwestern Medical Center at Dallas, IRB, Dallas, TX. Canada: IRB Services, Aurora, Ontario. Belgium: Universitair Ziekenhuis Gent, Commissie voor Medische Ethiek, Ghent. Denmark: De Videnskabsetiske Komiteer for Region Hovedstaden, Hillerød. Finland: Tukija, Valvira, Helsinki. Germany: Ethik-Kommission der Medizinischen Fakultät der Universität Duisburg-Essen, Nordrhein-Westfalen. Spain: Hospital Universitari Vall d'Hebron, Comité Ético de Investigación Clínica Barcelona, Barcelona. United Kingdom: NRES Committee London - City & East Bristol REC Centre, Whitefriars, Bristol Avon. Greece: National Ethics Committee, Athens Holargos. France: CPP Sud Mediterannée V, Nice. Italy: Comitato Etico Regione Lombardia – Istituto Carlo Besta, Milan. Netherlands: METC Brabant, Tilburg. The studies were conducted in accordance with the local legislation and institutional requirements. The participants provided their written informed consent to participate in this study.

## Author contributions

RJ: Writing – original draft. CT: Writing – review & editing. TM: Data curation, Writing – review & editing. JB: Data curation, Writing – review & editing. CZ: Data curation, Writing – review & editing. YD: Data curation, Formal analysis, Writing – review & editing. SA: Data curation, Writing – review & editing. JM: Data curation, Writing – review & editing.
