# Supplementary material for: Baseline demographics and disease characteristics of patients with episodic or chronic cluster headache: data from two phase 3 randomized clinical trials in Europe and North America
Source: Front Neurol. 2023 Dec 15;14:1293163. doi: 10.3389/fneur.2023.1293163 (PMC10756139; doi:10.3389/fneur.2023.1293163)
Supplement: Supplementary file 1 [file Data_Sheet_1.docx]

**Supplementary Materials**

**Supplementary Table 1. Baseline demographics and disease state characteristics collected at Visit 1**

|  | **ECH total^1^**  **N = 106** | **CCH total^2^**  **N = 237** | **Pooled total**  **N = 343** |
| --- | --- | --- | --- |
| Age, years |  |  |  |
| Mean (SD) | 46.4 (11.1) | 45.0 (10.9) | 45.4 (11.0) |
| Min, Max | 19.0, 65.0 | 18.0, 65.0 | 18.0, 65.0 |
| Sex, n (%) |  |  |  |
| Male | 88 (83.0) | 172 (72.6) | 260 (75.8) |
| Race, n (%) |  |  |  |
| Black or African American | 6 (5.7) | 2 (0.8) | 8 (2.3) |
| White | 90 (84.9) | 200 (84.4) | 290 (84.6) |
| Other | 10 (9.4) | 35 (14.8) | 45 (13.1) |
| Ethnicity, n (%) |  |  |  |
| Hispanic or Latino | 7 (6.6) | 33 (13.9) | 40 (11.7) |
| Not Hispanic or Latino | 86 (81.1) | 168 (70.9) | 254 (74.1) |
| Not applicable^a^ | 13 (12.3) | 36 (15.2) | 49 (14.3) |
| Body mass index, kg/m^2^ |  |  |  |
| Mean (SD) | 26.7 (4.7) | 26.4 (4.8) | 26.5 (4.8) |
| Min, Max | 19.6, 39.6 | 15.8, 39.8 | 15.8, 39.8 |
| Region, n (%) |  |  |  |
| Europe | 70 (66.0) | 196 (82.7) | 266 (77.6) |
| North America | 36 (34.0) | 41 (17.3) | 77 (22.5) |
| Duration of CH illness,^b^ years, mean (SD) | 16.8 (11.3) | 8.0 (7.1) | 10.7 (9.5) |
| Attacks in last 7 days, n (%) | 80 (75.5) | 237 (100.0) | 317 (92.4) |
| Lifetime suicidal ideation prior to screening,^c^ n (%) | 14 (13.2)^d^ | 55 (23.2) | 69 (20.1) |
| Lifetime suicidal behavior prior to screening,^e^ n (%) | 1 (0.9)^f^ | 9 (3.8)^g^ | 10 (2.9) |

CCH, chronic cluster headache; CH, cluster headache; C-SSRS, Columbia-Suicide Severity Rating Scale; ECH, episodic cluster headache; Max, maximum; Min, minimum; N, number of intent-to-treat patients with nonmissing demographic measures; n, number of patients within each specific category; SD, standard deviation.

^a^ ‘Not applicable’ response indicates the patient did not provide ethnicity details.

^b^ Defined as (informed consent date - first CH medical history start date + 1)/365.25. N = 103 in the ECH study; data were missing for one patient in the placebo group and for two in the galcanezumab group. N = 235 in the CCH study; data were missing for one patient in each treatment group. N = 338 in the pooled total.

^c^ Includes a 'yes' answer to any of the five suicidal ideation questions (categories 1-5) on the C‑SSRS.

^d^ Of the patients only reporting suicidal ideation, the most severe ideation reported by placebo‑treated patients was active suicidal ideation with any methods (no plan) and, for galcanezumab‑treated patients, nonspecific active suicidal thoughts.

^e^ Includes a 'yes' answer to any of the five suicidal behavior questions (categories 6-10) on the C‑SSRS.

^f^ A single patient reported preparatory acts or behavior and an aborted attempt.

^g^ N = 236; data were missing for one patient in the placebo group. Of these nine patients, three reported preparatory acts or behavior, four reported an aborted attempt, two reported an interrupted attempt, and five reported an actual, non-fatal attempt; some patients reported more than one type of suicidal behavior.

**Supplementary Table 2. Use of acute treatments during prospective baseline phase**

|  | **ECH total**  **N = 106** | **CCH total**  **N = 237** | |
| --- | --- | --- | --- |
| Patients using acute treatments, n (%) |  |  | |
| Sumatriptan SC | 56 (52.8) | 149 (62.9) | |
| Oxygen | 54 (50.9) | 140 (59.1) | |
| Oral/intranasal triptans | 23 (21.7) | 66 (27.9) | |
| Acetaminophen/paracetamol or NSAIDs | 40 (37.7) | 39 (16.5) | |
| Number of acute treatments/week, mean (SD) | | |  |
| Sumatriptan SC | 9.1 (9.1) | 9.3 (10.4) | |
| Oxygen | 15.2 (48.5) | 16.4 (35.9) | |
| Oral/intranasal triptans | 4.8 (5.3) | 6.6 (7.0) | |
| Acetaminophen/paracetamol or NSAIDs | 8.0 (13.3) | 6.9 (11.0) | |

CCH, chronic cluster headache; ECH, episodic cluster headache; NSAIDs, nonsteroidal anti‑inflammatory drugs; SC, subcutaneously.

**Supplementary Table 3. Alcohol, caffeine, nicotine, and tobacco use**

| n (%) | **ECH total**  **N = 106** | **CCH total**  **N = 237** |
| --- | --- | --- |
| Current alcohol use^a^ | 65 (61.3) | 111 (46.8) |
| Current caffeine use^b^ | 96 (90.6) | 220 (92.8) |
| Current nicotine use^c^ | 4 (3.8) | 9 (3.8) |
| Current tobacco use^d^ | 55 (52.4)^e^ | 149 (62.9) |

CCH, chronic cluster headache; ECH, episodic cluster headache; N, number of intent-to-treat patients with nonmissing habit measures; n, number of patients within each specific category

^a^ Alcohol use included beer, wine, and spirits.

^b^ Caffeine use included coffee, espresso, and tea.

^c^ Nicotine use included e-cigarettes, nicotine patches, and nicotine gum.

^d^ Tobacco use included cigarettes, cigars, smokeless (pinches) tobacco, and pipes.

^e^ N = 105 in the ECH study; data on tobacco use were missing for one patient in the galcanezumab group.

**Note:** There were no significant differences between placebo- and galcanezumab-treatment groups in either study.

**Supplementary** **Table 4. Pre-existing conditions reported by ≥5% of either patients with ECH or CCH at baseline**

| **n (%)** | **ECH total**  **N = 106** | **CCH total**  **N = 237** |
| --- | --- | --- |
| Patients with ≥1 pre-existing condition | 106 (100.0) | 237 (100.0) |
| Hypertension | 7 (6.6) | 25 (10.6) |
| Insomnia | 11 (10.4) | 24 (10.1) |
| Gastroesophageal reflux disease | 11 (10.4) | 12 (5.1) |
| Depression | 6 (5.7) | 23 (9.7) |
| Anxiety | 6 (5.7) | 19 (8.0) |
| Hypercholesterolemia | 8 (7.6) | 16 (6.8) |
| Hypothyroidism | 6 (5.7) | 18 (7.6) |
| Back pain | 6 (5.7) | 17 (7.2) |
| Seasonal allergy | 5 (4.7) | 14 (5.9) |
| Migraine | 1 (0.9) | 13 (5.5) |
| Asthma | 6 (5.7) | 9 (3.8) |
| Drug hypersensitivity | 6 (5.7) | 5 (2.1) |
| Headache | 6 (5.7) | 1 (0.4) |

CCH, chronic cluster headache; ECH, episodic cluster headache; N, number of intent-to-treat patients; n, number of patients within each specific category.

**Supplementary Reference List**

1. Goadsby PJ, Dodick DW, Leone M, Bardos JN, Oakes TM, Millen BA, et al. Trial of galcanezumab in prevention of episodic cluster headache. New Engl J Med. (2019) 381(2):132-41. doi: 10.1056/NEJMoa1813440.

2. Dodick DW, Goadsby PJ, Lucas C, Jensen R, Bardos JN, Martinez JM, et al. Phase 3 randomized, placebo-controlled study of galcanezumab in patients with chronic cluster headache: results from 3-month double-blind treatment. Cephalalgia. (2020) 40(9):935-48. doi: 10.1177/0333102420905321.
